# The Role of Street Medicine and Mobile Clinics for Persons Experiencing Homelessness: A Scoping Review

**DOI:** 10.3390/ijerph21060760

**Published:** 2024-06-12

**Authors:** Rebekah A. Kaufman, Mahwish Mallick, Jarvis Thanex Louis, Mollie Williams, Nancy Oriol

**Affiliations:** 1 Harvard Medical School, 200 Longwood Ave, Boston, MA 02115, USA; rebekah_kaufman@hms.harvard.edu (R.A.K.); thanex_louis@hms.harvard.edu (J.T.L.); nancy_oriol@hms.harvard.edu (N.O.); 2 Massachusetts General Hospital, 55 Fruit Street, Boston, MA 02114, USA; 3 Beth Israel Deaconess Medical Center, 330 Brookline Ave, Boston, MA 02215, USA

**Keywords:** mobile health, street medicine, persons experiencing homelessness

## Abstract

Introduction: An estimated 5800 to 46,500 lives are lost due to homelessness each year. Experiencing homelessness and poor health are cyclically related, with one reinforcing the other. Mobile programs, which include vehicles that travel to deliver care, and street medicine, the act of bringing care to spaces where PEH live, may play a role in alleviating this burden by providing trusted, affordable, and accessible care to this community. Methods: We conducted a scoping review of peer-reviewed literature on the role of mobile clinics and street medicine in providing care for PEH by searching PubMed, Embase, and Web of Science on 10 August 2023. Articles from 2013 to 2023 specific to programs in the United States were included. The protocol was developed following the PRISMA-ScR guidelines. The primary outcome was the role of mobile programs for persons experiencing homelessness. Results: A total of 15 articles were included in this review. The descriptive findings emphasized that street medicine and mobile clinics provide primary care, behavioral health, and social services. The utilization findings indicate that street medicine programs positively impact the health system through their ability to defer emergency department and hospital visits, providing financial benefits. The comparative findings between mobile programs and office-based programs indicate current successes and areas for improvement. Discussion: Mobile clinics and street medicine programs that serve PEH provide a wide range of services. While more significant structural change is needed to address healthcare costs and housing policies in the United States, mobile clinics and street medicine teams can improve healthcare access and the healthcare system.

## 1. Introduction

An estimated 5800 to 46,500 lives are lost in the United States due to homelessness each year [1]. Notably, this range denotes potential underreporting of homelessness and large differences between the known and estimated trends [1,2,3]. However, in 2023, it is known homelessness increased by more than 12%, reaching a record high of 653,104 estimated persons experiencing homelessness (PEH) [4,5]. Significant disparities exist in who is at risk of experiencing homelessness in the United States, with Black, Hispanic, and American Indian or Alaskan Native people having an increased risk of homelessness [6,7]. Additionally, sexual and gender minorities (SGM) are more likely to experience homelessness [8]. Racial and ethnic disparities stem in part from historical practices such as redlining, but current discrimination against racial and ethnic minorities and SGM plays a role as well [7,9]. These alarming trends need attention, especially from a healthcare viewpoint [4,10].

Homelessness, a lack of fixed or stable housing, is a major social determinant of health [10,11,12]. There are many barriers that prevent PEH from accessing healthcare, resulting in a cyclic pattern, as experiencing homelessness and poor health are reinforced and compounded by one another [11,13]. Poor health, mentally and physically, may result in missed work, leading to financial risk of eviction, possible loss of health insurance, and worsening health conditions [11,13].

PEH have higher mortality rates due to infectious diseases, cardiovascular disease, accidental injury, suicide, homicide, and substance abuse disorder (SUD) [14,15]. Additionally, 60% of PEH do not have health insurance, and 73% reported at least one unmet healthcare need in the last year [16]. The most frequently cited reasons for an unmet need were the inability to afford care and a lack of health insurance [16]. However, other barriers to accessing care include transportation, distrust of the healthcare system, and stigma [17,18,19]. The current healthcare delivery system does not adequately address the structural barriers that prevent PEH from receiving healthcare [10,16,17]. In fact, it was not until 2004 that general guidelines were created for physicians who worked with PEH, and it was in 2018 that the Street Medicine Institute created more specific international guidelines about primary care for PEH [17]. Overcoming these barriers is necessary to provide care for PEH.

In this review, we examine two interrelated models of low-barrier healthcare: (1) mobile clinics, which provide care in vehicles, and (2) street medicine, which provides care directly in the environments frequented by PEH, such as shelters and encampments [20,21]. It is important to note that street medicine teams may also operate mobile clinics, and conversely, mobile clinics can serve as platforms for street medicine, reflecting a degree of operational overlap between the two models [22,23]. It has been reported that both types of mobile programs improve healthcare access for marginalized groups, lead to favorable health outcomes, mitigate transportation barriers, and reduce healthcare costs [20,24]. However, limited research exists on the use of mobile programs for PEH, and we are not aware of any other scoping reviews. This review aims to close the knowledge gap on the role of mobile programs for PEH.

## 2. Methods

### 2.1. Protocol

The protocol for this review follows the PRISMA-SCR guidelines [25]. The protocol has not been shared publicly and is not registered anywhere online but can be provided by the corresponding author.

### 2.2. Eligibility Criteria

We included peer-reviewed articles that examined the role and potential impact of mobile clinics and street medicine programs in delivering healthcare to PEH, including original research, reviews, and editorials. Conference abstracts, reports, and dissertations were excluded. Articles published from 2013 to 2023 in English about mobile programs in the United States were included. Only articles from the past ten years were included for relevant information regarding the current social, political, and financial landscape.

### 2.3. Population, Concept, and Context

The population of focus for the review was PEH. The concept guiding this review was to synthesize information to deepen our understanding of the ways that mobile programs serve PEH, the roles they play, and the services they provide [25]. The context was various locations within United States, chosen due to its distinctive challenges and comparatively poor outcomes, particularly in its care for PEH [26,27].

### 2.4. Search Methods

#### Information Sources and Search

PubMed, Embase, and Web of Science were searched for relevant information on 10 August 2023. Articles published from 2013 to 2023 were included to ensure the information was current. Relevant keywords included “mobile clinic”, “street medicine”, and “homelessness”. The complete PubMed search strategy is available in Appendix A as Item 1.

### 2.5. Selection of Sources of Evidence

#### Study Selection

Screening articles for inclusion was a stepwise process. References from PubMed, Web of Science, and Embase were imported into Covidence, where duplicates were removed. Titles and abstracts were screened independently by two reviewers (R.K. and M.M.). Subsequently, full texts were screened independently for inclusion by two reviewers (R.K. and M.M.). Disagreements were discussed between the reviewers, and a conclusion was made by referring to the protocol. Articles that did not fit the inclusion criteria were excluded.

### 2.6. Data Charting, Data Items, and Synthesis of the Results

The included articles were exported into EndNote version 20.5 and Excel version 2108 from Covidence independently by one reviewer (R.K.). The data were extracted into Excel and included information about author name, article title, journal of publication, and year of publication. The type of study (e.g., original research, editorial, case study, analysis, or commentary) and funding were also collected. The included articles were reviewed. Information about the types of services provided and the potential impact of mobile programs were pulled from the articles and grouped together in Excel. This process was iterative, and thematic groupings evolved as the articles were reviewed.

### 2.7. Critical Appraisal

Given the descriptive nature of many of the included studies, a critical appraisal of individual sources was not performed. Additionally, conducting a critical appraisal of individuals sources is an optional component of scoping reviews [25].

## 3. Results

### 3.1. Selection of Sources of Evidence

**Figure 1:**
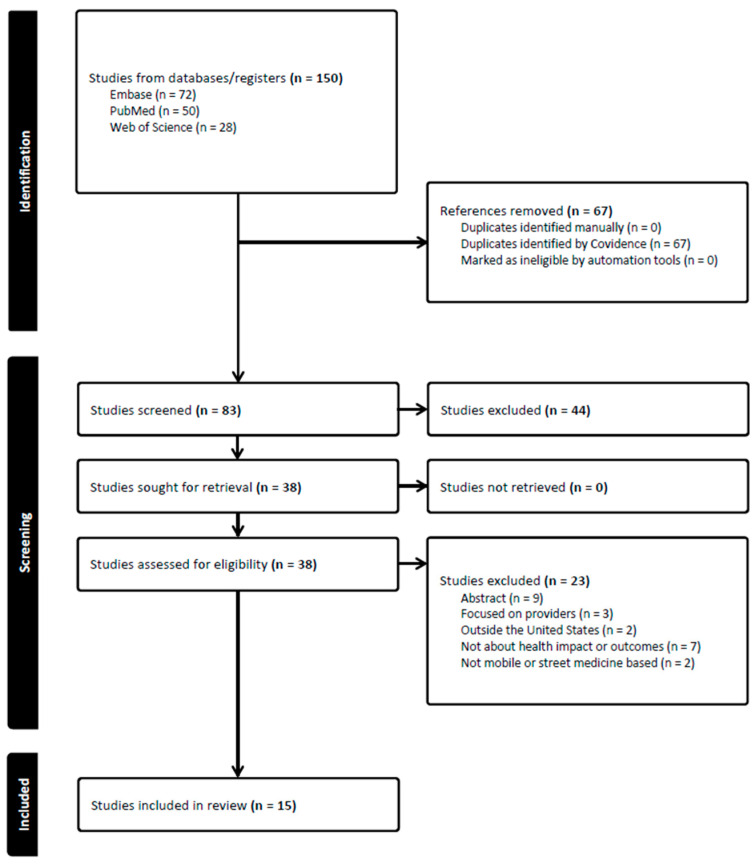
PRISMA 2020 Flow Diagram. Adapted from [28] and Covidence provided diagram with modifications.

The screening and study selection process is available in Figure 1. 

### 3.2. Characteristics of Sources of Evidence

Information about each included article, including program geographic information, year of publication, article type, and the program’s focus, if available, is presented in Table 1. See Appendix Table A1 for results on the individual sources of evidence.

### 3.3. Synthesis of the Results

**Figure 2:**
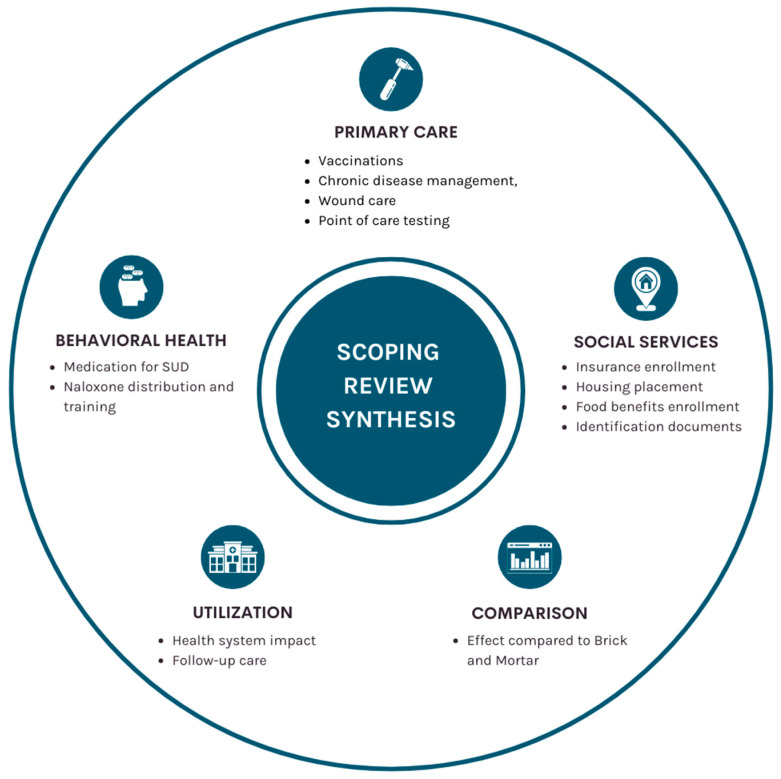
Services provided by and role of mobile programs for PEH.

A total of 15 studies examined mobile programs’ role in providing care for PEH. Most (*n* = 10) of the cities with programs included in this review are classified as urban by the US Census Bureau [29]. One program (*n* = 1) described itself as serving “rural mid-Atlantic communities.” Three papers (*n* = 3) reported on an overall state. Lastly, one article (*n* = 1) was non-specific to location, describing the nationwide health system impact. Most of the included articles reported descriptive data about the services provided by mobile programs (*n* = 13). From the descriptive data, most (*n* = 8) reported on behavioral health services, followed by primary care services (*n* = 7) and social services (*n* = 3). In addition to providing descriptive data, one study (*n* = 1) conducted qualitative interviews of PEH who engaged with their team. Three (*n* = 3) papers reported on the health system utilization impact of mobile programs for PEH. Four articles (*n* = 4) compared a mobile program to an office-based location. Notably, some of the included manuscripts reported a combination of descriptive data, comparative data, and health system utilization data. A full list of the groupings and their components can be found in Figure 2.

### 3.4. Behavioral Health

Eight papers reported on behavioral health services, including general psychiatric care and substance use disorder (SUD) care.

Many mobile programs deliver behavioral health services for PEH. In California, 25 street medicine programs provided behavioral health services, serving 9682 unique patients in 2021 alone [30]. An individual mobile clinic in Massachusetts (UMass Memorial Medical Center) has cared for 1121 individuals, with over 4567 encounters [31]. Programs such as these play a role in the diagnosis of mental health disorders, continuing treatment for conditions, the distribution of psychiatric medications, substance use disorder treatment, medication management for assisted treatment (MAT) with buprenorphine, naloxone distribution, and counseling services [30,31,32,33,34,35].

Several programs provided substance use disorder treatment, including MAT [31,32,36]. Notably, buprenorphine prescription was reported by programs [31,32,36]. In a retrospective chart review, a buprenorphine program administered by a street medicine team successfully engaged patients and decreased the barriers to access [32]. The UMass Memorial Medical Center mobile clinic reported prescribing buprenorphine to 330 individuals, and their naloxone distribution reported 74 rescue attempts [31]. In fact, naloxone distribution was reported by multiple organizations [31,37]. One street medicine team also reported providing and evaluating education on how to use naloxone [37]. Pre- and post-tests were given to evaluate the efficacy of naloxone use training [37]. Of the 194 participants, the average score increase was 2.02 from the pre-test, indicating the street team had a statistically significant impact (*p* < 0.0001) and effectively provided educational training [37].

### 3.5. Primary Care

Seven papers discussed primary care services, including preventative screening, acute care, and chronic disease management.

Mobile programs offer core primary care services and preventative screenings. A qualitative study of a street medicine program found that PEH highly valued the connection to a primary care provider [35]. General medical consultations and health screenings, including blood pressure screening, blood glucose testing, vision testing, hepatitis C diagnosis, and HIV testing, are reported in the literature [34,38,39]. Additionally, the management of chronic conditions such as hypertension, diabetes, chronic obstructive pulmonary disease, asthma, clotting issues, and orthopedic pain are offered [34,35]. Disease management and prevention are often provided through medication dispensation, vaccination, and wound care [38,39,40]. In fact, one “suitcase clinic” in Virginia saw 269 unique clients, had 1200 visits, and provided about $12,000 worth of free medication [34]. The vaccinations reported by mobile programs include flu, COVID-19, Hepatitis A, and Mpox vaccines [39]. Two case studies reported on wound care by street medicine teams in Miami [40,41]. One case study discussed the management of pyoderma gangrenosum, a type of ulcerating dermatosis, by a street medicine team [41] Another case study described in detail the use of street medicine for wound care [40]. This case followed the care of a soft tissue infection in the leg that developed after an incident where the individual was hit by a car [40]. Decreasing barriers to transportation, particularly in an acute injury setting, can create improved access to care [40]. Point-of-care testing and partnerships with clinical laboratories play a role in mobile programs’ ability to provide services and full-spectrum quality primary care [38,42]. While the program resources vary currently in Los Angeles, point-of-care testing by street medicine teams includes glucose meters but is expanding to include urine and blood testing [42].

### 3.6. Social Services

Three papers reported social services, such as housing placement, insurance enrollment, and assistance in obtaining identification documents.

A vital component of many mobile programs is providing social services [33,35,38,43]. The most reported social services were insurance enrollment, housing placements, and connection to food benefits such as SNAP [33,35,38]. Notably, one street medicine team helped clients obtain identification so they could engage in social programs and receive benefits [35]. Qualitative interviews of PEH in Austin, TX, reported the social services provided by a “suitcase clinic” are one of its greatest benefits [43]. In Hawaii, qualitative data also report that a street medicine team improved healthcare navigation and reduced hesitance in accessing care [35]. More so, overall quality of life improved after engaging with the street medicine team as a result of being connected to housing [35].

### 3.7. Utilization

Three papers reported on health system impact and mobile programs’ impact on system utilization.

Street medicine positively impact health systems through decreased ED visits and hospitalizations. Notably, street medicine programs have decreased ED visits and hospitalizations by 75% and 66%, respectively [33]. In a cohort of PEH who were Medicare/Medicaid-eligible in Southern California, 87% of their care costs were related to the emergency department, hospital visits, or skilled nursing facility visits [33]. When modeling a 15% decrease in these visits, there would be a cost savings of $9000 per patient every year (based on the annual cost of care calculated to be $69,000 in this cohort) [33]. In addition to fiscal benefits, street medicine can improve follow-up retention rates [38]. A program in Los Angeles, CA, which identifies PEH during inpatient appointments and consults them improved their follow-up with future appointments [38]. Of the PEH who received a consult, 70% followed up with the street team after discharge [38]. In comparison, only 27% of PEH who did not receive a consult returned to an affiliated clinic within 6 months [38]. Hospitals may not have the resources to maintain contact with patients, and street medicine can help improve retention rates in a transitionary care role [38,40].

### 3.8. Comparison to Brick and Mortar

Four papers quantitatively or qualitatively compared mobile programs to office-based locations.

Street medicine programs have been evaluated and compared to traditional clinic settings, with varying results [32,44]. Compared to an office location, a pilot buprenorphine program run by a street medicine team in San Francisco, CA, had lower retention rates [32]. At 12 months, the street medicine group retained 26% of patients, while the office retained 61% [32]. Notably, the office-based program excluded patients with other substance use disorders and severe mental illnesses [32]. A “street psychiatry” team in Connecticut prescribing buprenorphine found that half of the enrolled patients continued for three months, and one-third continued for at least six months [36]. This is in line with other mobile programs but lower than the office-based retention rates [36]. More recently, a matched cohort compared the healthcare utilization of PEH after visiting a mobile addiction clinic or fixed site in Boston, MA [43]. Patients who visited the mobile clinic within its first year of use were compared to patients with SUD who visited a fixed site during the same time [43]. No statistically significant differences in health system utilization were found following the visits [43]. Qualitative research found that PEH receiving care at a “suitcase clinic” located within a church felt its inclusiveness and combination of both healthcare and social service agencies were vital to engagement [44]. In fact, this was reported as an advantage when compared to patients interviewed from a shelter clinic [44]. In this case, the shelter clinic had a transportation advantage; however, negative encounters with other individuals were reported because the space was less secure [44]. Drug use in the shelter clinic was also reported as a negative because it was difficult for patients who were sober to be in the environment [44].

## 4. Discussion

### 4.1. Summary of Evidence

Mobile programs serving PEH provide multidisciplinary services, including behavioral health, primary care, and social services [30,34,35]. Mobile programs have provided thousands of PEH with free behavioral health and primary care services, signifying the ability of mobile programs in bridging care gaps [30,31,34]. Notable services provided by mobile programs include free vaccinations, free medication dispensation, buprenorphine treatment, naloxone distribution, and assistance obtaining identification documents [31,32,34,36,37,39]. While there is less quantitative information comparing established mobile programs to their office-based counterparts, particularly outside of substance treatment, the qualitative data suggest that mobile-based programs develop and maintain strong patient relationships and improve their quality of life [32,35,36,44]. Lastly, mobile programs also have a positive impact on the health system and decrease ED visits and hospitalizations while increasing cost savings [33].

These findings align with the scope of mobile programs internationally. Research from low- and middle-income countries indicates that mobile health programs may improve healthcare access for children who are underserved and experiencing homelessness [45]. Additionally, qualitative research in Canada reports that PEH feel less stigma when receiving care from street medicine providers, echoing the qualitative findings from the United States [46,47]. Other examples include India and Brazil, where mobile clinics play significant roles in healthcare for PEH [48,49]. In Delhi, India, street medicine teams conducted consults with more than 16,000 individuals, diagnosing conditions such as upper respiratory infections, gastritis, tinea, and helminthiasis [48]. In Brazil, street clinics employ diverse teams that provide harm reduction services [49].

While limited research exists on mobile programs, the existing literature describes the ability of these services to provide care for PEH. The findings from this paper summarize preliminary information about the role and potential impact of mobile programs for PEH. These results can both inform potential or existing mobile programs and promote the efficacy of mobile health in improving healthcare access for underserved populations. More research is needed to quantitively compare established mobile programs using matched controls to office-based locations, particularly for primary care and social services. Further research and data are also needed to better understand the true number of people experiencing homelessness, as certain populations, such as women, experience “hidden homelessness” at higher rates [9,50]. A deeper understanding of the extent of homelessness can highlight the importance of health services and the types of care needed [9,50]. Not only could further research provide insight into the potential impact of and areas of improvement for mobile programs but it could also be used to develop and update guidelines for mobile programs serving PEH [17]. While primary care guidelines exist for PEH, there are no guidelines for behavioral health providers, which could improve the behavioral health services provided by mobile programs [17]. More extensive discussions about structural change are needed to advance health equity. Without addressing the lack of shelter through practices such as housing first, healthcare and public health professionals will always remedially serve PEH [51]. Promoting policy changes that value healthcare and housing as human rights would have a significant effect on the health of PEH [51].

### 4.2. Limitations

This study had three main limitations. First, there was a lack of qualitative research about the experiences of PEH who receive care from mobile programs in the United States. Understanding the narratives of PEH can greatly improve services and advocate for the potential importance of mobile programs. Second, there was a lack of quantitative data comparing established mobile programs to office locations outside of behavioral health. Lastly, the exclusion of gray literature is a limitation, as more information may be found in these sources.

## 5. Conclusions

Mobile clinics and street medicine programs that serve PEH provide a wide range of services [30,33,34,38]. These findings demonstrate the importance of mobile programs that prioritize PEH. While more significant structural change is needed to address healthcare costs and housing policies in the United States, mobile clinics and street medicine teams improve both access to healthcare and the healthcare system in general [51,52].

## Figures and Tables

**Table 1:** Characteristics of included studies.

Article Title	Published Year	Article Type	Authors	Program Type	Location if Available	Focus
The Case for Mobile “Street Medicine” for Patients Experiencing Homelessness.	2022	Editorial	Lynch KA; Harris T; Jain SH; Hochman M	Street Medicine	United States	Business Case for Street Medicine
Mpox Case Reports in an Urban Homeless Population and a Proof of Concept for a Street-Based Mobile Mpox Vaccination Clinic.	2023	Case Studies	Zeien J; Vieira J; Hanna J; Surendra L; Stenzel J; Ramirez A; Miller C; Rosales C	Street Medicine	Phoenix, AZ	Vaccination; Mpox
A Study on the Efficacy of a Naloxone Training Program.	2021	Original Research	Beauchamp GA; Cuadrado HM; Campbell S; Eliason BB; Jones CL; Fedor AT; Grantz L; Roth P; Greenberg MR	Street Medicine	Pennsylvania	Naloxone Distribution and Training
From the hospital to the streets: Bringing care to the unsheltered homeless in Los Angeles.	2021	Original Research	Feldman BJ; Kim JS; Mosqueda L; Vongsachang H; Banerjee J; Coffey CE Jr; Spellberg B; Hochman M; Robinson J	Street Medicine	Los Angeles, CA	Care Follow-Up
Low barrier buprenorphine treatment for persons experiencing homelessness and injecting heroin in San Francisco.	2019	Original Research	Carter J; Zevin B; Lum PJ	Street Medicine	San Francisco, CA	MAT; Buprenorphine
The dynamics of providing street medicine to a geographically diverse homeless population in Hawaii.	2023	Original Research	Maxwell D; Thomas J; Plassmeyer M	Street Medicine	Hawaii	Qualitative Experiences
Soft tissue infection and follow-up for an unsheltered patient: the role of Street Medicine providers in bridging gaps in care.	2023	Case Report	Rasul TF; Morgan O; Elkhadem A; Henderson A	Street Medicine	Miami, FL	Wound Care
Understanding the primary health care experiences of individuals who are homeless in non-traditional clinic settings.	2022	Original Research	Ramirez J; Petruzzi LJ; Mercer T; Gulbas LE; Sebastian KR; Jacobs EA	“Suitcase Clinic”	Austin, TX	Qualitative Experiences
Health Care Utilization among Homeless-Experienced Adults Who Were Seen by a Mobile Addiction Health Clinic in Boston, Massachusetts: A Quasi-Experimental Study.	2023	Original Research	Fine DR; Joyce A; Chang Y; Lewis E; Weinstock K; Wright J; Gaeta J; Song Z; Baggett TP	Mobile Clinic	Boston, MA	Addiction Health/Opioid Use Treatment
Point-of-Care Testing to Support a Street Medicine Program in Caring for the Homeless.	2021	Professional Insights	Chambliss AB; Johnson G; Robinson J; Banerjee J; Feldman BJ	Street Medicine	Los Angeles, CA	Point of Care Testing: Glucose Meters
A mobile addiction service for community-based overdose prevention.	2023	Case Reports	Pepin MD; Joseph JK; Chapman BP; McAuliffe C; O‘Donnell LK; Marano RL; Carreiro SP; Garcia EJ; Silk H; Babu KM	Mobile Clinic	Worchester, MA	MAT, Methadone, Buprenorphine, or Naloxone Distribution
Resource-Limited Management of Presumptive Pyoderma Gangrenosum in an Unsheltered Patient.	2022	Case Reports	Rasul TF; Mathew M; Anderson JD; Bergholz DR; Henderson A	Street Medicine	Miami, FL	Chronic Disease Management
Behavioral Health Care Delivery Through Street Medicine Programs in California.	2023	Original Paper	Su KY; Feldman BJ; Feldman CT; Saluja S; Coulourides Kogan AM; Cousineau MR	Street Medicine	California	Mental Health and Substance Use Treatment
Addressing Health Care Needs in the Homeless Population: A New Approach Using Participatory Action Research	2018	Research Article	Kiser, T; Hulton, L	“Suitcase Clinic”	Rural Mid-Atlantic Community	Urgent Care, Chronic Disease Management, Psychiatric Medication Distribution, Dental, Podiatric
Low-Barrier Buprenorphine Treatment for People Experiencing Homelessness.	2023	Frontline Report	Gibson CL; Lo E	Street Medicine	New Haven, CT	MAT; Buprenorphine

## Data Availability

This review is based on published literature. The References section provides citations of the sources and data.

## Appendix A

Item 1. Pub Med Search Strategy

((“mobile clinic”[All Fields] OR “mobile clinic”[All Fields] OR “street medicine”[All Fields]) AND (“ill housed persons”[MeSH Terms] OR (“ill housed”[All Fields] AND “persons”[All Fields]) OR “ill housed persons”[All Fields] OR “homeless”[All Fields] OR “homelessness”[All Fields])) AND (y_10[Filter])

Translations

homelessness: “ill-housed persons”[MeSH Terms] OR (“ill-housed”[All Fields] AND “persons”[All Fields]) OR “ill-housed persons”[All Fields] OR “homeless”[All Fields] OR “homelessness”[All Fields]

**Table A1:** Results on individual sources of evidence.

Authors	Population	Funding Source	Services and Role	Sector of Impact
Lynch 2022	PEH	Healthcare in Action Medical Group	Nationwide data	Utilization, Behavioral Health, and Social Services
Zeien 2023	PEH	None declared	Vaccination	Primary Care
Beauchamp 2021	PEH	Dorothy Rider Pool Health Care Trust Research and Development Award for Transformational Excellence	Naloxone distribution and training	Behavioral Health
Feldman 2021	PEH	Street medicine team funded by the Department of Family Medicine at an academic institution	In-patient follow-up, medical consultations, and housing placement	Primary Care, Social Services, and Utilization
Carter J 2019	PEH	None declared for this publication, but first author reports funding from UCSF Primary Care Addiction Medicine Fellowship	Buprenorphine treatment and retention rates	Behavioral Health and Comparison to Brick and Mortar
Maxwell 2023	PEH	Castle Foundation	Quantitative impact of primary care and social services	Primary Care, Social Services, and Behavioral Health
Rasul 2023	PEH	None declared	Wound care and follow-up	Primary Care and Utilization
Ramirez 2022	PEH	BSM PRIDE Institute SRP funding from NHLBI (R25HL105444) and T32HL140290 Post-doctoral Training in Promoting Health Equity in Cardiovascular Disease at the University of Texas at Austin	Qualitative experiences	Comparison to Brick and Mortar
Fine 2023	PEH	Kraft Center for Community Health, the National Institutes of Health (K12DA043490), the Division of General Internal Medicine at Massachusetts General Hospital (MGH), the MGH Research Scholars Program, and the National Institutes of Health (DP5-OD024564). Did not play a role in analysis or publication.	Addiction care utilization	Comparison to Brick and Mortar
Chambliss 2021	PEH	None declared	Point-of-care testing	Primary Care
Pepin 2023	PEH	MA Department of Public Health/Bureau of Substance Addiction Services, the Kraft Center for Community Health, the Overdose Prevention Fund at UMass Memorial Health/UMass Chan Medical School, the RIZE Foundation, and TD Bank	Opioid overdose prevention	Behavioral Health
Rasul 2022	PEH	None declared	Wound care	Primary Care
Su 2023	PEH	Statewide California Electronic Library Consortium	Data from street medicine programs in California	Behavioral Health
Kiser 2018	PEH	None declared	Urgent care, chronic disease management, psychiatric medication distribution, dental, podiatric	Primary Care and Behavioral Health
Gibson 2022	PEH	None declared	Buprenorphine treatment	Behavioral Health and Comparison to Brick and Mortar
